# Effect of calf-raise training on rapid force production and balance ability in elderly men

**DOI:** 10.1152/japplphysiol.00539.2016

**Published:** 2017-06-01

**Authors:** Ryoichi Ema, Shunsuke Ohki, Hirokazu Takayama, Yuji Kobayashi, Ryota Akagi

**Affiliations:** ^1^Graduate School of Engineering and Science, Shibaura Institute of Technology, Saitama, Japan;; ^2^Research Fellow of Japan Society for the Promotion of Science, Tokyo, Japan;; ^3^College of Systems Engineering and Science, Shibaura Institute of Technology, Saitama, Japan; and; ^4^Institute for Education and Student Services, Okayama University, Okayama, Japan

**Keywords:** rate of torque development, plantar flexion, home-based, single-leg standing, electromyography

## Abstract

This study examined whether home-based, high-speed calf-raise training changes the rate of torque development (RTD) during plantar flexion contractions and balance performance in elderly men. Thirty-four healthy elderly men (73 ± 5 yr) were randomly assigned to a training or control group (*n* = 17 in each group). The subjects in the training group completed 8 wk (3 times/wk) of home-based bilateral calf-raise training using body mass. Before and after the intervention, RTD during plantar flexion contractions and center-of-pressure (COP) displacement during single-leg standing were measured. Surface electromyographic amplitude of the triceps surae and tibialis anterior during the strength and single-leg standing was measured. Clinical magnitude-based inferences were used to interpret the training effect, with the smallest worthwhile effect assumed to be 0.2 of the baseline SD. The peak RTD increased 21% (90% confidence limits, ±19%) relative to the control group, which was accompanied by corresponding changes of the medial gastrocnemius (MG) and soleus (SOL) activations. The effect on COP displacement was possibly trivial (0%, ±13%), whereas substantial reduction in the MG (−19%, ±15%) and SOL (−25%, ±13%) activations during standing was observed. Our findings indicate that calf-raise training at home, performed without special equipment or venue, induces a substantial increase in the plantar flexors’ rapid force-generating capability and triceps surae activations. Although the training effect on standing balance performance was not substantial, observed changes in the triceps surae activations during standing are expected to contribute to future balance performance improvement.

**NEW & NOTEWORTHY** Calf-raise training with the intent to move rapidly, without special equipment or venue, induces an improvement of explosive plantar flexion force, which is attributable to neuromuscular rather than musculotendinous adaptations. Although the training effect on balance performance was trivial, we found a sign of improvement (i.e., neuromuscular adaptations during standing). In conclusion, functional neuromuscular capacity can be enhanced by home-based calf-raise exercise in elderly men, which may protect against mobility loss with aging.

## Abstract (precis)

Calf-raise training with the intent to move rapidly, without special equipment or venue, induces an improvement of explosive plantar flexion force, which is attributable to neuromuscular rather than musculotendinous adaptations. Although the training effect on balance performance was trivial, we found a sign of improvement (i.e., neuromuscular adaptations during standing). In conclusion, functional neuromuscular capacity can be enhanced by home-based calf-raise exercise in elderly men, which may protect against mobility loss with aging.

age-related decline of explosive strength of the skeletal muscles (2, 32, 45) can be a significant limiting factor in daily activities (9). Explosive strength is often evaluated as the rate of force development (RFD), which is defined as the slope of the time force/torque curve during the initial phase of isometric contraction from a low or resting level (1, 3, 5). Izquierdo et al. (30) observed that the RFD during isometric squat was lower in elderly than in young people and that a lower RFD was related to impaired balance performance. In addition, it was suggested that an age-related decrement of plantar flexion RFD leads to a high risk of falling (36). These findings indicate the importance of preventing the reduction in RFD for elderly people so as to maintain their functional capacity in daily life. In particular, it is possible that an increase in the plantar flexion RFD would result in improved balance performance and thus help to reduce the risk of falling.

Resistance exercise involving explosive muscle contraction (i.e., as fast and forcefully as possible) is effective for enhancement of RFD in elderly (14, 17, 44) as well as young (6a, 47, 52) people. The exercise load in those previous studies was relatively high [8–12 repetition maximum load (17, 44, 52), 75–80% of 1 repetition maximum load (14), and ≥80% (6a) or >90% (47) of maximal voluntary contraction (MVC) force or torque]. In contrast, at least for plantar flexion, training with low exercise load (30–40% of 1 repetition maximum load) at high speed induced an increase in RFD in young individuals (23). In addition, power training at low load improved balance performance in elderly people (43). With the consideration that the muscularity of the plantar flexors in elderly people is reduced compared with young people (7, 24) and that relatively low external load moved at a high speed can involve high forces of agonist muscles, we expected that training with low exercise load, such as calf-raise exercises using body mass at high speed (i.e., with the intent to move rapidly), would induce an increase in their plantar flexion RFD and balance performance. Clarification of the training response induced by body mass-based exercise would provide beneficial information regarding effective training programs for elderly people, because such exercises can be performed regardless of location and without special equipment and might reduce injury risks while facilitating continuation of the exercise. The purpose of the current study was to examine the effect of home-based, high-speed calf-raise training for the plantar flexors on rapid force production and balance performance in elderly men.

## METHODS

### Subjects

Thirty-four healthy elderly men (73 ± 5 yr, 165 ± 7 cm, 64 ± 9 kg, means ± SD) volunteered to join the study. The study participants were assigned to a training or control group (*n* = 17 in each group) by stratified randomization in advance so as to match the age, height, and body mass between the two groups. The mean height in the training group was less than that in the control group by 2.5 cm (a small difference), but differences in age (1 yr) and body mass (0.6 kg) were trivial. All of the participants were functionally independent in daily life, whereas some of them participated in walking [*n* = 10 (training group) and *n* = 11 (control group)], ground golf [*n* = 9 (training group) and *n* = 8 (control group)], table tennis [*n* = 3 (training group) and *n* = 0 (control group)], softball [*n* = 2 (training group) and *n* = 2 (control group)], and/or tennis [*n* = 0 (training group) and *n* = 2 (control group)], once or twice per week, and others were sedentary [*n* = 2 (training group), *n* = 2 (control group)]. None of the participants performed resistance exercises. This study was approved by the Ethics Committee of the Shibaura Institute of Technology. The subjects were informed of the purpose and potential risks of the study and provided written informed consent before participation.

### Experimental Design

The subjects in the training group performed 8 wk of home-based bilateral calf-raise training and maintained their usual diet. The time of day for exercising was not controlled to maximize compliance with the exercises and to diminish the possibility of dropout from the study. The subjects in the control group were requested to continue their normal daily activities and eating habits throughout the same period. Before and after the intervention (2–3 days after the last training session in the training group), maximum and explosive plantar flexion strength; balance performance; muscle activation of the medial gastrocnemius (MG), lateral gastrocnemius (LG), soleus (SOL), and tibialis anterior (TA) during strength and balance measurements; muscle thickness of the triceps surae; and muscle architecture (muscle thickness, pennation angle, and fascicle length) of MG were determined. The test measurements were performed on the pivot leg, defined as the opposite leg to that used for kicking a ball.

### Training Protocol

The subjects in the training group conducted bilateral calf-raise exercises at home, three times per week for 8 wk, with 1 or 2 days between the training sessions. One session of the training comprised 3 sets of 10 repetitions with a 2-min rest between sets. From a standing position with both feet on flat ground, the subjects raised their heels (concentric phase) as fast as possible and dropped them at a moderate speed (~2 s), with the knees straight. One cycle of this action was performed every 5 s. The touching of a wall or chair was allowed during the training sessions so as to prevent falls during exercise. The first training session was performed in the laboratory with the aid of explanation and feedback by an examiner. Midway through the intervention (i.e., after 4 wk from the beginning of the training program), the subjects visited our laboratory again to be checked as to whether the training protocol was being performed correctly. During the intervention period, each subject was asked to note on a form the following instructions: *1*) please check the box of the day when you conducted the calf-raise exercises, *2*) in each set, if you completed 10 repetitions, please check the box on a form; otherwise, please enter the number of repetitions that you did. The subjects submitted the forms twice (i.e., midway through the intervention and after the intervention). In addition, an examiner sometimes called to verify the forms. As a result, all subjects in the training group completed the program correctly (i.e., 3 times per week, 3 sets of 10 repetitions) without any injuries or diseases.

### Strength Testing

**Fig. 1.**
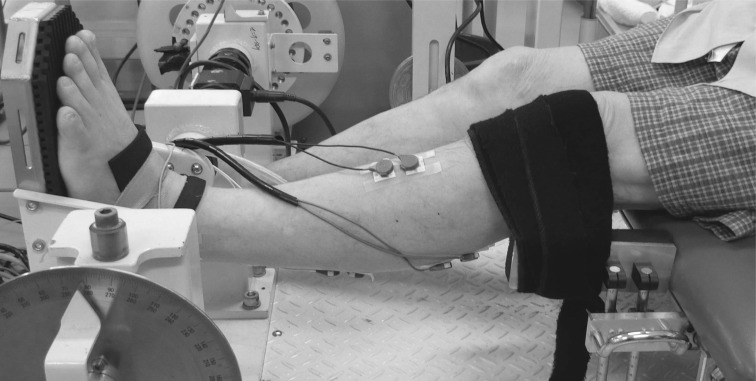
Experimental setup for strength testing.

**Fig. 2.**
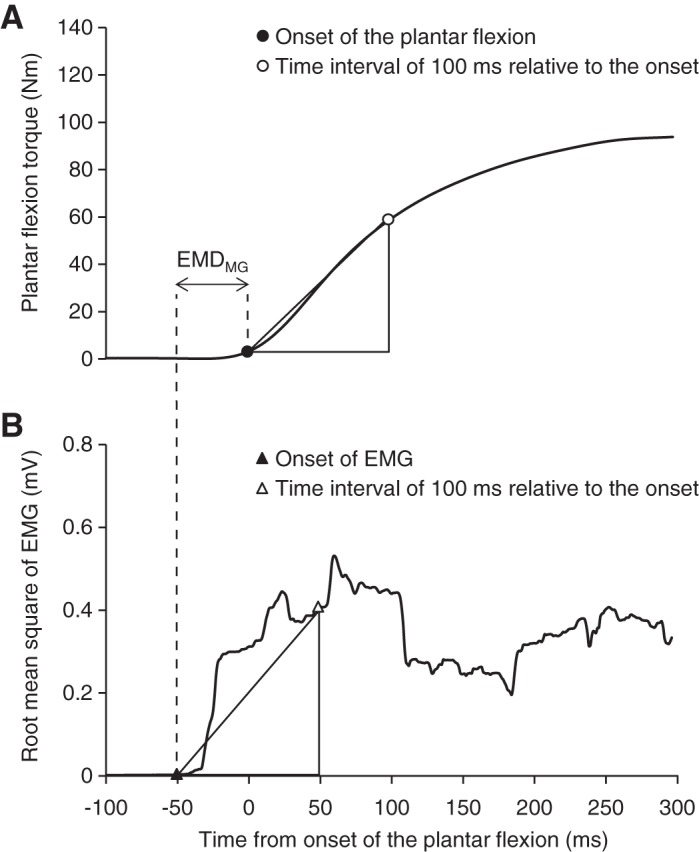
Examples of torque (*A*) and smoothed electromyography (EMG) signals (*B*; a 50-ms moving root mean square window) of the medial gastrocnemius during maximal voluntary isometric plantar flexion. Time = 0 corresponds to the onset of plantar flexion. Onsets of the plantar flexion/EMG signal are denoted by black circle/triangle, and time intervals of 100 ms relative to onsets are indicated by white circle/triangle. Rate of torque development (RTD) was defined as the slope of the time-torque curve over time intervals of 0–10 to 0–250 ms from the onset of plantar flexion. The peak value of the first derivative of the time-torque curve was defined as RTD_peak_. Rate of EMG rise (RER) was defined as the slope of the time-smoothed EMG curve over time intervals of 0–10 to 0–250 ms from the onset of EMG signals. Determinations of RTD and RER over the time interval of 0–100 are indicated as examples. The difference between the onset of plantar flexion and EMG signals was defined as electromechanical delay of the medial gastrocnemius (EMD_MG_).

**Fig. 3.**
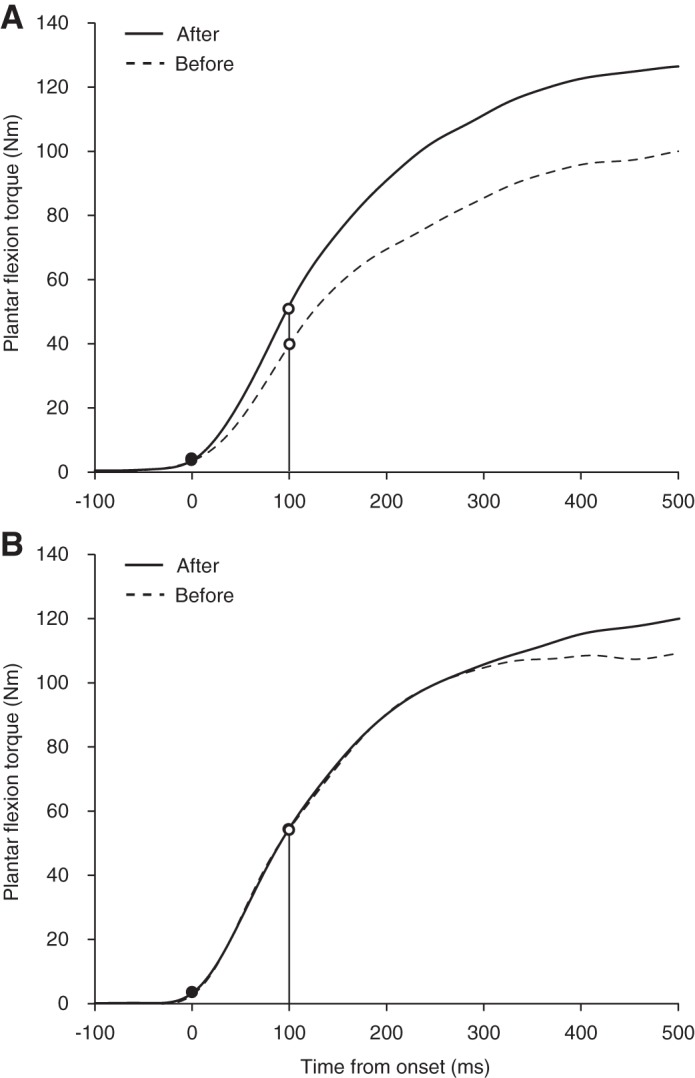
Examples of time-torque curve of a subject in the training (*A*) and control (*B*) groups before (dotted lines) and after (solid lines) the 8-wk training period. Onset of plantar flexion is denoted by black circles. Vertical lines and white circles indicate time intervals of 100 ms relative to the onset of plantar flexion as an example of 1 time point.

Isometric plantar flexion MVC was measured with a specially customized dynamometer equipped with a torque transducer (TD200; Kubota, Osaka, Japan) (33) (Fig. 1). The subject sat on the bench of the dynamometer (hip joint angle = 80°, knee joint angle = 0°, and ankle joint angle = 0°; anatomical position = 0°), while securing the knee and foot on the bench and footplate with nonelastic straps to prevent knee flexion and heel raise during contractions. Care was taken to adjust the centers of rotation of the dynamometer and ankle joint. We carefully matched the subject’s relative position on the dynamometer between the measurements before and after the intervention. After several submaximal plantar flexion contractions with the intent to perform rapidly as a warm-up, the subject was encouraged to perform plantar flexion as fast and forcefully as possible and to maintain plantar flexion for 3 s. The contractions were conducted three times with 1-min rests, and verbal encouragement was provided during all contractions. The torque signals were acquired at 1,000 Hz with an analog-to-digital converter (PowerLab16/35; ADInstruments, New South Wales, Australia) and transferred to a computer. In addition, to normalize the electromyographic (EMG) values of TA to those from the MVC trial, isometric dorsiflexion with maximal effort was also performed. With the use of a fourth-order zero-phase lag Butterworth filter, the torque signal was low-pass filtered at 15 Hz (1). The peak value of each torque signal was defined as MVC torque. The rate of torque development (RTD) was defined as the slope of the filtered time-torque curve over time intervals of 0–10 to 0–250 ms from the onset of plantar flexion, respectively (3, 5) (Figs. 2 and 3). The peak value of the first derivative of the time-torque curve (RTD_peak_) was also determined using derivative function (time period: 0.001 s). The onset of plantar flexion was defined as the instant when plantar flexion torque exceeded the baseline by 2.5% of MVC torque (1, 46). We assessed RTD at several time intervals, because different physiological factors are reported to be related to early and later phases of RTD (3, 22) and because a previous longitudinal study observed phase-specific changes in RTD after resistance training (5). In addition, RTD normalized to MVC torque (normalized RTD) was calculated (1, 5). The trial in which the highest RTD_peak_ or MVC torque was observed was used for the later analyses of RTD and MVC torque, respectively. The means of the coefficient of variation (CV) and intraclass correlation coefficients type 1.1 [ICC (1,1)] of the highest and second-highest values were 10.1% and 0.930 for RTD_peak_ and 3.2% and 0.977 for MVC torque, respectively. Day**-**to-day repeatability of the measurements (for elderly men, *n* = 10, 74 ± 6 yr) was also evaluated. The means of CVs and ICCs (1,1) were 10.6% and 0.914 for RTD_peak_ and 6.1% and 0.938 for MVC torque, respectively.

### Balance Performance Measurement

Static balance performance with single-leg standing was evaluated similarly to a previous study (20). The subjects stood barefoot with one leg (pivot leg) on the platform of a center-of-pressure (COP) tracking instrument (T.K.K.5810; Takei Scientific Instruments, Niigata, Japan) for 30 s with their eyes open. During the standing, the participants fixed their gaze on a visual target mounted on a wall, 2 m in front of them, located at eye height above the floor. They were asked to hold their arms at their sides and to lift the opposite foot (kicking foot) with the knee flexed at ~90°. Three trials were performed with sufficient rests between the trials. If the subjects could not keep the raised foot up or the supporting leg left on the platform, then the trial was ended. The COP signals were sampled at 20 Hz and stored on a personal computer without being lowpass filtered. Before the subject stood on the platform, we confirmed no COP displacement on the raw data (i.e., high-frequency noise contamination seems unlikely). The data of the first 5 s from the beginning of the onset were eliminated to prevent the effect of postural changes on the COP trajectory. Thereafter, the COP displacement was determined for the time period corresponding to 5–30 s, and the total COP displacement divided by the above time period (i.e., 25 s) was defined as COP speed. The minimum of COP speed among the trials in which the subject could remain standing for 30 s was used for further analysis. When the subject could never stand for 30 s, the trial in which the subject could stand longest was selected for the analyses, and the path length was determined from 5 s after onset until 3 s before the end of the trial. Regarding the day-to-day repeatability, the means of CV and ICC (1,1) of the COP speed on two different days (for elderly men, *n* = 10) were 5.4% and 0.907, respectively.

### EMG Measurement

Surface EMG signals were acquired from MG, LG, SOL, and TA of the pivot leg during the plantar flexion strength and balance performance measurements. The muscle belly and fascicle longitudinal directions were confirmed using real-time B-mode ultrasonography (ACUSON S2000; Siemens Medical Solutions, Malvern, PA), with a probe perpendicular to the skin for identifying the fascicles. After skin shaving, rubbing with sandpaper, and cleaning with alcohol, bipolar Ag/AgCl surface electrodes (F-150M; size adjustment by cutting to 10 × 20 mm, 20 mm interelectrode distance) with high-pass filtering at 5 Hz, using a bioamplifier system (MEG-6108; Nihon Kohden, Tokyo, Japan), were placed at 40% (MG and TA) and 30% (LG) of the lower-leg length, determined as the length from the popliteal crease to the lateral malleolus. For SOL, electrode placement was ~5 cm distal from the level of 40% of the lower-leg length, but there were interindividual variations due to the variability of the superficial region of SOL. To match the electrode placement between before and after the intervention, the subjects were requested to keep some marks on their skin by tracing after taking a bath every day; these were recorded in the test before the intervention, and photographs of the electrode placement were taken during the test. The EMG signals were recorded at a sampling frequency of 1,000 Hz and stored in a personal computer after analog-to-digital conversion (PowerLab16/35; ADInstruments). Regarding the analyses of EMG signal data on strength measurements, EMG signals were smoothed with a 50-ms moving root mean square (RMS) window (1). The rate of EMG rise (RER) of the triceps surae muscles during MVC trials was determined as the slope of the smoothed time–EMG curve from EMG onset to 10–250 ms (Fig. 2). The threshold of EMG onset was set at means + 3 SDs from the baseline (means and SD were calculated at rest over a 200-ms time window). In addition, the electromechanical delay of MG (EMD_MG_) was determined as the difference in time between onsets of plantar flexion torque and EMG amplitude of MG (Fig. 2). The EMD_MG_ at the anatomical position (i.e., joint angle in the current strength measurement) is less affected by slack in the Achilles tendon (41), and the value is associated with the Achilles tendon stiffness (50), which is one of the predictors of the plantar flexion RFD (50). Although these analyses were performed automatically using MATLAB software (MathWorks, Natick, MA), onsets of EMG signals were visually confirmed because the automatic detection is not always accurate (48). In addition, the RMS values of EMG signals (RMS-EMGs) during MVC trials were calculated over a 0.5-s period around the maximal torque. With respect to the EMG signals during balance performance measurements, RMS-EMG during single-leg standing was calculated from high-pass-filtered EMG signals for the period corresponding to the COP displacement determination and normalized to those recorded during MVC trials.

### Ultrasonographic Measurement

The muscle thickness of the triceps surae and pennation angle of MG were measured using B-mode ultrasonography (for muscle thickness of the triceps surae, SSA-770 Aplio 80; Toshiba Medical Systems, Tochigi, Japan; for MG architecture, ACUSON S2000, Siemens Medical Solutions) with a 60-mm width linear-array probe. The muscle thickness of the triceps surae was determined during quiet standing at 30% of the lower-leg length from the popliteal crease to the lateral malleolus (40). The muscle thickness and pennation angle of MG were measured in a sitting position on the seat of the dynamometer with the muscles relaxed at the same joint angles used for strength testing. With respect to MG measurements, special care was taken to match the measurement regions and fascicles before and after the intervention as follows. First, the measurement region was assigned along the lower-leg length. Second, the mediolateral width of MG was determined over the skin by identifying the boundaries between MG and LG and between MG and the tibia. Lastly, the measurement regions were determined as 40% of the lower length from the popliteal crease to lateral malleolus and 40% of the mediolateral width from the boundary between MG and tibia. The muscle thickness of MG was defined as the distance between superficial and deep aponeuroses at the center of each image, and the pennation angle was determined as the angle between the fascicle and deep aponeurosis (42). The fascicle length of MG was estimated from the muscle thickness and sine component of the pennation angle (31). These analyses were conducted using ImageJ software (National Institutes of Health, Bethesda, MD). The measurement was conducted two times for muscle thickness of the triceps surae and three times for muscle architecture of MG, and mean values of the repeated measures were used for further analyses. The means of CVs and ICCs (1,2) or (1,3) of the repeated measurements were 0.6% and 0.996 for muscle thickness of the triceps surae, 3.8% and 0.982 for muscle thickness of MG, 2.8% and 0.973 for MG pennation angle, and 4.1% and 0.954 for MG fascicle length, respectively. The repeatability of ultrasound measurements on 2 different days was evaluated (for elderly men, *n* = 10). The means of CVs and ICCs (1,1) were 1.5% and 0.920 for muscle thickness of the triceps surae, 5.6% and 0.813 for muscle thickness of MG, 5.1% and 0.872 for pennation angle of MG, and 4.6% and 0.847 for fascicle length of MG, respectively.

### Physical Activity during the Intervention Period

Given that it was difficult to match the exercise habits perfectly between the two groups, physical activity during the intervention period for 8 wk was monitored using a triaxial accelerometer (Actimarker EW4800; Panasonic Electric Works, Osaka, Japan). The accuracy of such measurement has been confirmed (51). The subject was asked to wear the device during daily life, except for time spent in the bath or sleeping. The mean magnitude of physical activity (minutes/day) was determined at three levels using the concept of metabolic equivalents (METs): low (1.5–2.9 METs), moderate (3.0–5.9 METs), and vigorous intensities (>6.0 METs), respectively (20, 39).

### Statistical Analysis

All data were log transformed before analyses. For ease of interpretation, the data are presented as means ± SD of raw data, unless noted otherwise. Because of disconnection of the EMG cable, EMG signals of LG could not be recorded from three subjects (2 in the training group and 1 in the control group) at measurement before the intervention. Clinical magnitude-based inferences were performed to assess the magnitude of training-induced changes in variables using a published spreadsheet (27). The approach determines the training effect as unclear if the confidence interval includes values that are substantial in some beneficial and harmful sense; otherwise, the effect is characterized with probabilistic terms. Regarding COP speed and RMS-EMG of TA during MVC, there were moderate group differences at baseline, and thus an adjustment for baseline was performed in the spreadsheet. We chose three values of the covariate for adjustment of the training effect: overall mean and highest and lowest values of the control group. Standardized difference in the mean change was calculated as the difference in mean change between the two groups divided by between-subject SD at baseline, and 0.2 of between-subject SD was assumed to be the smallest worthwhile effect. According to the suggestions of Hopkins et al. (28), the level of confidence interval was set at 90%. The qualitative probabilistic terms were assigned as follows (28): 25–75%, possibly; 75–95%, likely; 95–99.5%, very-likely; >99.5%, most likely. If chance of benefit and harm were >50% and >1.5%, respectively, then the training effect was interpreted as clinically unclear (25). The percent effect of the training was determined as the difference in the mean change between the two groups via back transformation. In addition, an SD of individual responses to training in percent units for main outcomes was calculated from a spreadsheet (27). The thresholds for interpretation of the standardized mean change/difference were 0.2, 0.6, and 1.2 for small, moderate, and large, and the value was halved for interpreting individual responses to training (26).

## RESULTS

### Strength

**Fig. 4.**
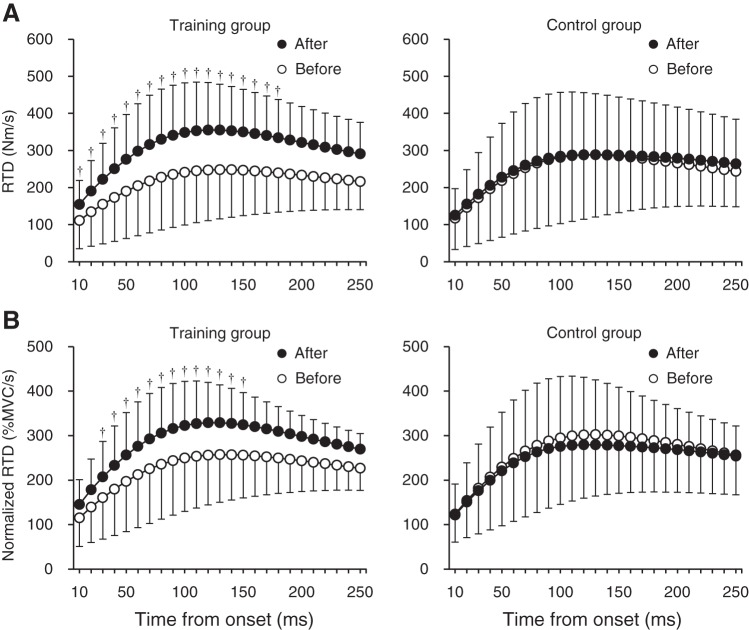
The rate of torque development (RTD; *A*) and RTD relative to maximal voluntary contraction (MVC) torque of the plantar flexion (normalized RTD; *B*) in the training (*left*) and control (*right*) groups before and after the intervention. The RTD was defined as the slope of the filtered time-torque curve over time intervals of 0–10 to 0–250 ms from the onset of plantar flexion. Symbols denote clearly substantial effects: †, likely beneficial change. The changes in RTD at 240 and 250 ms were clear and at least possibly trivial. Data are presented as means ± SD of raw data.

There were substantial increases in absolute RTD (percent effects, 34–106%) and normalized RTD (51–104%), except for at some phases (clinically unclear: absolute values at 190–230 ms and normalized values at 10–20 and 160–250 ms; possibly trivial: absolute values at 240 and 250 ms), with the observed effects as small to moderate (Fig. 4). With respect to RTD_peak_ and plantar flexion MVC torque (Table 1), there were likely (RTD_peak_) and possibly (MVC torque) beneficial effects of training. The SDs of individual responses to training in percent units were moderate for RTD_peak_ (28% and 90% confidence limits, ±24%) and unclear for MVC torque (−6%; ±16%) because of large uncertainty. A likely substantial decrease (−14%; 90% confidence limits, ±14%) was observed in dorsiflexion torque (training group, from 21 ± 5 to 20 ± 7 Nm; control group, from 21 ± 7 to 22 ± 7 Nm).

**Table 1.** Strength, balance performance, and muscle architectural values before the intervention, those changes, and difference in the changes between the training and control groups

	Training Group (*n* = 17)		Control Group (*n* = 17)		
	Before	Change, %	Before	Change	Difference in the Mean Change (±90% CL)
*Strength*					
RTD_peak_	360 ± 160 N·m·s^−1^	36 ± 37	420 ± 190 N·m·s^−1^	12 ± 22	21 (±19)↑†
MVC torque	99 ± 28 N·m	14 ± 18	100 ± 27 N·m	7 ± 20	7 (±11)↑*
*Balance performance*					
COP speed	24 ± 9 mm·s^−1^	−12 ± 21	20 ± 6 mm·s^−1^	−12 ± 25	0 (±13)
*Muscle architecture*					
Triceps surae					
Muscle thickness	66.1 ± 5.9 mm	1.9 ± 5.4	64.7 ± 4.6 mm	0.4 ± 1.3	1.5 (±2.3)
Medial gastrocnemius					
Muscle thickness	18.2 ± 2.7 mm	−2.2 ± 9.3	17.3 ± 2.3 mm	−1.0 ± 9.1	−1.3 (±5.1)
Pennation angle	18.6 ± 2.0°	−0.1 ± 9.8	18.5 ± 2.4°	−0.5 ± 12.9	0.4 (±6.3)
Fascicle length	57.5 ± 9.7 mm	−2.1 ± 11.0	55.2 ± 8.3 mm	−0.6 ± 12.5	−1.6 (±6.4)

Values are means ± SD unless noted elsewhere. Change and difference in the mean change were calculated via back transformation with a published spreadsheet, and the data of COP speed were determined after adjustment for baseline. The changes in muscle architecture were not substantial (i.e., absolute value of the standardized difference in the mean change was below 0.2) but clear and at least possibly trivial for muscle thickness of the triceps surae and pennation angle of the medial gastrocnemius and possibly harmful for muscle thickness and fascicle length of the medial gastrocnemius. CL, confidence limit; RTD_peak_, peak value of rate of torque development; MVC torque, peak value of generated torque at maximal voluntary plantar flexion trial; COP, center of pressure. ↑, a substantial increase, and symbols denote clearly substantial effects.

* Possibly beneficial change.

† Likely beneficial change.

### Balance Performance

The magnitude of observed training effect on COP speed was interpreted as possibly trivial (Table 1) when adjustment for baseline was performed with overall mean, whereas the threshold was trivial but possibly harmful because of low chance of benefit with adjustment to highest (−3%; 90% confidence limits, ±25%) and lowest (3%; ±19%) values of the control group. The SD of individual responses to the training in percent unit was estimated to be unclear because of large uncertainty (−12%; ±18% with the 3 adjustment values).

### EMG Variables

#### RER.

**Fig. 5.**
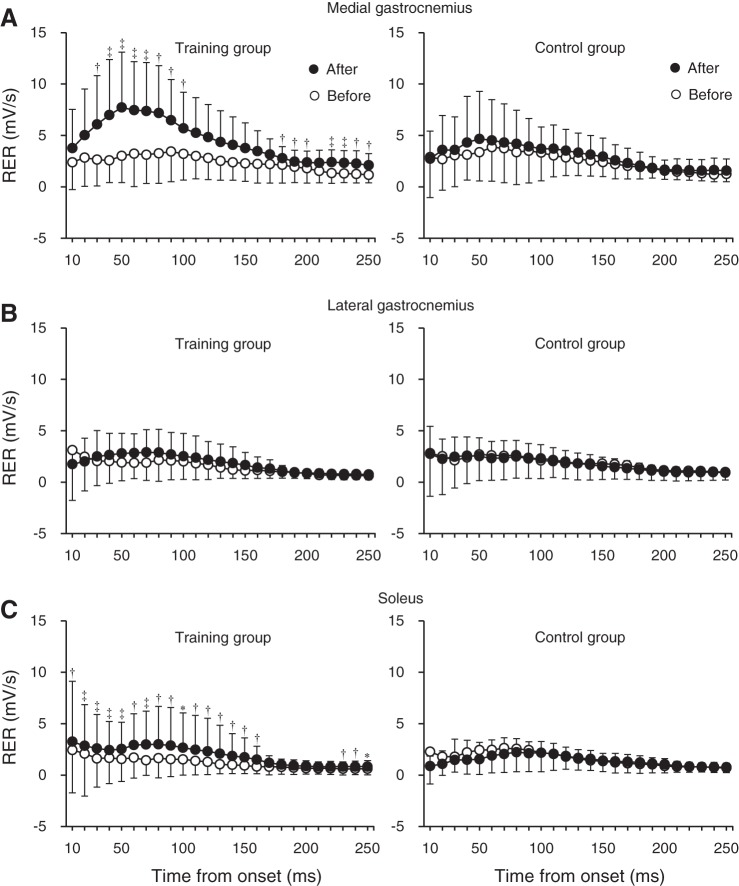
The rate of electromyography rise (RER) of the medial gastrocnemius (*A*), lateral gastrocnemius (*B*), and soleus (*C*) in the training (*left*) and control (*right*) groups before and after the intervention. The RER was defined as the slope of the smoothed time-electromyogram curve over time intervals of 0–10 to 0–250 ms from the onset of electromyogram. Symbols denote clearly substantial effects: *, possibly beneficial change; †, likely beneficial change; ‡, very-likely beneficial change. The changes in RER of the lateral gastrocnemius were not substantial (i.e., absolute value of the standardized difference in the mean change was below 0.2) but clear and at least possibly trivial/harmful at 10–40, 80, 100–130, and 180–250 ms. Data are presented as means ± SD of raw data.

Figure 5 shows RERs of the agonist plantar flexor muscles. There were substantial increases in RERs of MG at 30–100, 180–200, and 220–250 ms (percent effects, 58–206%) and SOL at 10–160 and 230–250 ms (47–391%), whereas those at other regions were possibly trivial or clinically unclear. No substantial changes were shown in the effects of RERs of LG or TA during plantar flexion contractions at all phases.

#### EMD_MG_.

The EMD_MG_ was 63 ± 21 ms (before) and 63 ± 16 ms (after) in the training group and 67 ± 21 ms (before) and 66 ± 25 ms (after) in the control group. The effect of the training was possibly trivial (6%; 90% confidence limits, ±28%).

#### RMS-EMG during MVC trials.

There was a very-likely substantial effect on RMS-EMG of MG (36%; 90% confidence limits, ±28%), with the magnitude of effect being moderate. The values in the training group were 0.38 ± 0.20 mV (before) and 0.49 ± 0.21 mV (after) and were 0.36 ± 0.14 mV (before) and 0.35 ± 0.13 mV (after) in the control group. The likely beneficial effects were observed on RMS-EMGs of LG (20%; ±23%) and SOL (27%; ±20%). The effects on those of TA during dorsiflexion MVC (−18 through −1%) and during plantar flexion MVC as an antagonist (23–33%) were possibly harmful, with the threshold as trivial to small, except when that during dorsiflexion MVC was adjusted to lowest baseline value of the control group (25%; ±84% “clinically unclear”).

#### RMS-EMG during single-leg standing.

**Fig. 6.**
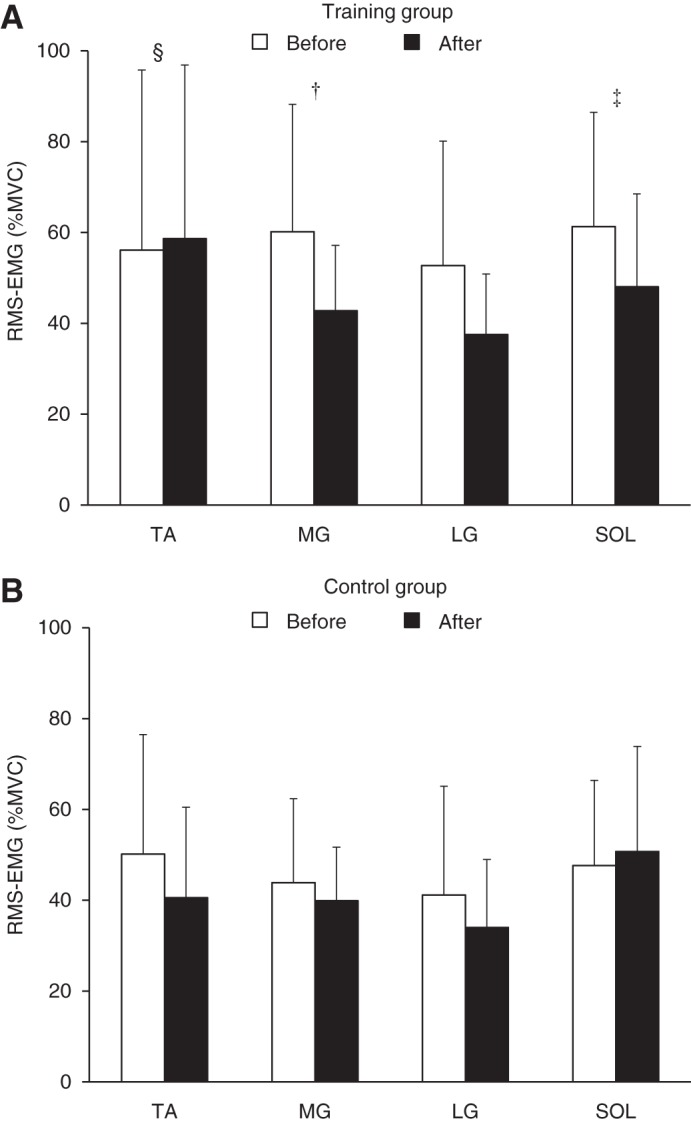
The root mean square values of electromyogram (RMS-EMG) during single-leg standing relative to those recorded at maximal voluntary contraction (MVC) in the training (*A*) and control (*B*) groups before (open bars) and after (black bars) the intervention. Symbols denote clearly substantial effects: †, likely beneficial change; ‡, very-likely beneficial change; §, likely harmful change. The change in lateral gastrocnemius (LG) was clear and at least possibly trivial. TA, tibialis anterior; MG, medial gastrocnemius; SOL, soleus. Data are presented as means ± SD of raw data.

The normalized RMS-EMGs of each muscle are shown in Fig. 6. The normalized RMS-EMGs of MG (−19%; 90% confidence limits, ±15%) and SOL (−25%; ±13%) substantially decreased (beneficial effect), as small to moderate effects, whereas that of TA (31%; ±40%) increased (harmful effect), with the threshold as small. In contrast, the effect on RMS-EMG of LG (−10%; ±25%) was possibly trivial.

### Muscle Architecture

The values of architectural parameters are shown in Table 1. No substantial effects were shown in the variables, whereas the trivial effects were clear: possibly trivial for muscle thickness of the triceps surae and pennation angle of MG and possibly harmful for muscle thickness and fascicle length of MG. The SDs of individual responses to training in percent units were estimated to be 5.2% (90% confidence limits, ±1.8%) for muscle thickness of the triceps surae; 1.9% (±8.0%) for muscle thickness of MG; −7.4% (±9.2%) for pennation angle of MG; and −5.3% (±9.9%) for fascicle length of MG. The threshold of the SD was large for muscle thickness of the triceps surae, whereas unclear for other variables because of large uncertainties.

### Physical Activity during the Intervention Period

The magnitudes of physical activity during the training or control period were as follows: at light intensity, 242.5 ± 70.8 min/day (training group) and 233.4 ± 52.2 min/day (control group); at moderate intensity, 44.3 ± 28.7 min/day (training group) and 37.7 ± 21.4 min/day (control group); at vigorous intensity, 0.9 ± 3.4 min/day (training group) and 0.3 ± 0.4 min/day (control group). There were not any clear group differences in the magnitude of physical activity. The differences were 3% (90% confidence limits, ±15%) at low intensity, −15% (±50%) at moderate intensity, and 4% (±40%) at vigorous intensity.

## DISCUSSION

The current study demonstrated that without special equipment or venue, high-speed calf-raise training for 8 wk induced an improvement in both absolute and normalized values of plantar flexion RTD in elderly men. Because the normalized RTD reflects the capability of a subject to develop force rapidly, regardless of his maximum force capacity, and because muscle activation (i.e., RER) of the triceps surae was also increased without substantial changes in the triceps surae musculature, the present results suggest that the rapid force-generating capability of the triceps surae was improved by the intervention. In contrast, the change in static balance performance with single-leg standing was trivial, according to clinical magnitude-based inferences. However, muscle activations of MG and SOL during single-leg standing were substantially reduced after the intervention, which may result in better balance performance. The findings indicate that the calf-raise training used here is effective for improving RTD of the plantar flexion and exhibits neuromuscular adaptations that can contribute to balance performance improvement.

The increases in RER of the muscles trained in the current study are in line with previous studies (1, 4, 8, 18). Folland et al. (22) showed that the variability of RFD at an early phase (≤75 ms from the onset) was mainly explained by the variability of neural factors of agonist muscles, whereas the variability of MVC force was related to the RFD at a later phase. In addition, neural factors largely contributed to RFD relative to MVC force, irrespective of phase (22). The absolute and normalized RTDs were clearly increased, except for those at some later phases (Fig. 4). The improvement of RERs of MG and SOL was notable at the early phase (likely to very-likely beneficial change) and also occurred at the later phase (Fig. 5). Improved neural drive at the early phase has been reported after explosive strength training but not after sustained-contraction strength training (6a, 47). Thus exercises with the intent to move rapidly seem important for increasing agonist muscle activations, especially at the early phase. Regarding MVC torque, the effect was clinically clear, but the magnitude of training effect (7%, possible beneficial change) was smaller compared with that of RTD_peak_ (21%, likely beneficial change). This is consistent with a previous finding (47) after explosive-type training (explosive strength improvement > maximal strength improvement), suggesting that it may be difficult to obtain a similar training effect in maximal strength to that in explosive strength after explosive-type training. The increase in MVC torque was accompanied by substantial increases in RMS-EMGs of the triceps surae during MVC. Moreover, in the training group, relative change in MVC torque was clearly correlated with relative changes in RMS-EMG of MG (*r* = 0.71; 90% confidence limits, ±0.23) and LG (*r* = 0.76; ±0.20) using Pearson’s product-moment correlation coefficient. Although we did not determine the M-wave amplitude before and after the intervention, it was reported that explosive-type training did not induce changes in M-wave amplitude of the agonist muscles (38). Taken together, neural adaptation mechanisms in the triceps surae appear to be responsible for the observed increase in absolute and normalized RTD and MVC torque after calf-raise training.

Other factors that may contribute to RTD increases are changes in the architectural and mechanical properties of the muscle and/or tendon (37). Regarding the effect of muscle architecture, previous studies demonstrated that changes in muscle architecture of agonist muscles were associated with changes in RTD (13, 21, 44). We failed to find substantial changes in the muscle thickness of the triceps surae or MG, MG pennation angle, or fascicle length after the training period. A recent study (6a) demonstrated that sustained contraction but not explosive strength training for 12 wk increased the muscle size of the agonist muscles. With the consideration of the current and previous studies (6a), it seems that quantitative adaptation of agonist muscles is relatively unlikely after explosive-type training. The lack of substantial change in the muscle thickness of the triceps surae suggests that no clear changes occurred in the pennation angles or fascicle lengths of LG or SOL, considering the positive associations between the magnitudes of muscle hypertrophy and changes in the two parameters (19). Thus there was less impact of muscle architecture of the triceps surae on the improvement of RTD in the current study. With respect to the contribution of tendon mechanical properties, there is a positive relationship between tendon stiffness and RFD (50), and a training-induced increase in tendon stiffness is accompanied by an increment of RTD (35). In the current study, there was no substantial change in EMD_MG_ after the training program. The EMD_MG_ is associated with the Achilles tendon stiffness (50), and a decrease of EMD_MG_ after resistance training paralleled an increase in the Achilles tendon stiffness (49). Moreover, it was suggested that high-intensity training is required to induce an increase in the tendon stiffness of agonist muscles (6, 34). These previous findings suggest a lack of clear change in the Achilles tendon stiffness following the present training program (i.e., low-intensity exercise using body mass). Accordingly, it is unlikely that the improvement of RTD of the plantar flexion resulted from changes in musculotendinous factors of the triceps surae in the current study.

There were clear (moderate) individual differences in the effect of training on RTD_peak_. Although the SD of individual responses in MVC torque was unclear, the threshold could have been moderate considering the uncertainty. Because the current exercise protocol was body-mass based, the variability of each value relative to body mass at baseline may be one of the factors that results in the variability of training responses. Indeed, in the training group, there were clear negative correlations between relative change in RTD_peak_ (*r* = −0.62; 90% confidence limits, ±0.27) or MVC torque (*r* = −0.76; ±0.19) and the values relative to body mass at baseline. These findings suggest that great responses on rapid and maximal plantar flexion force generations are expected to occur in individuals who have relatively low capability of producing plantar flexion force.

We failed to find a beneficial effect on COP speed, whereas substantial reduction of muscle activations of MG and SOL was observed. It has been shown that there was a positive correlation between COP displacement and magnitude of RMS-EMG of the lower leg in young and elderly individuals (11) and has been suggested that impaired postural stability (i.e., large COP speed in the present study) in elderly people could be due to excessive activation of the triceps surae (15). To explore the contribution of changes in muscle activations to change in COP speed with baseline adjustment to overall mean, change scores of RMS-EMG in each muscle were dealt with as covariate in the spreadsheet (27). As a result of adjusting to zero change of the covariate, the effect was reduced from 0.3 to 8% with adjustment for MG, 9% for LG, and 0.6% for SOL. Consistent with the results, there were clear relationships between relative change in COP speed and relative changes in RMS-EMGs of MG (*r* = 0.48; 90% confidence limits, ±0.33) and LG (*r* = 0.67; ±0.25) in the training group. Adjustment for changes in RTD was also performed, and reduction of the training effect was shown to be from 0.3 to 0.8–3% at 10–150 ms but was not observed at later phases. These results suggest that calf-raise training-induced adaptations of the triceps surae activations and explosive plantar flexion strength at early phases beneficially affect static balance performance. The reasons for the lack of clear change in COP speed after the intervention are unclear, but likely harmful change on TA activation during single-leg standing may diminish the training effect on COP speed. Indeed, the adjustment for change in TA activation increased the effect from 0.3 to −3%. In contrast, given the means and uncertainty of the effect on COP speed, benefit could be small to moderate. Moreover, individual responses to training were unclear (−12%; ±18%), but the threshold could have been moderate considering the uncertainty. The means and uncertainty of the SD of individual responses were not substantially different even if we determined the values without adjustment for baseline (−10%; ±18%), implying that the baseline data did not affect substantially interindividual variability of training response. The negative value may be partly due to sampling variation, but if we ignore the uncertainties in the means and SD, then the effect on individuals could reach borderline small–moderate. Taken together, the present calf-raise training may potentially contribute to recover from reduced balance performance in elderly individuals. To detect clear training effect on balance performance, further studies are required.

In the present study, there was a difference in body position and equipment between the calf-raise exercise (standing position) and strength testing (sitting position). Although the current findings corroborate previous results that explosive-type training improves RFD of the lower extremity (17, 33, 47, 52), training has been performed at high intensity in the previous studies, and several studies have used the same apparatus for training and RFD evaluation (33, 47). Thus specificity of the training and/or learning effects may be inherent in the results of previous studies, and the transferability of the strength from the training mode to other tasks remains questionable. The lack of RFD change in the control group in the previous studies cannot rule out the above possibilities, because the control group would have occasion to use the apparatus only twice (i.e., measurements before and after the intervention), but the training group would use the equipment during training, as well as for performance measurements. Blazevich (12) suggested that the intent to move rapidly is not a major factor for the improvement of RFD if there is some specificity with respect to the training and testing exercises, whereas the intent is important when the test exercise is not specific to the training program. The current results support the notion of Blazevich (12). We need to take into account these points when considering training programs for functional improvement in elderly people.

Calf-raise training using body mass at high speed does not require any special equipment or venue, thereby simplifying continuation of the exercise by elderly people. Therefore, the current exercise program can be applied to rehabilitation or training at home. Recovery from a decrease in RFD after disuse is attenuated in elderly people (29). This indicates that elderly people live their everyday lives with a large risk of falling after recovery from diseases and/or injuries. The prevention of decrement of the plantar flexion RFD is crucial, considering its association with maximum walking speed (16) and risk of falling (36). The current exercise protocol can be of great benefit to elderly individuals who have impaired functional ability in daily life and who have difficulty in exercising at high intensity.

A methodological consideration is possible foot movement during strength testing. Although we fixed the subject’s foot on the plate of the dynamometer very tightly, slight foot movements, accompanied by changes of ankle joint angle, may have occurred during contractions. It is likely that such movement would induce larger muscle shortening of the triceps surae during isometric contractions, resulting in lower RTD at a time point. In addition, a small number of contractions and the use of the same contractions to assess RTD and MVC torque were limitations of the methods. These points may have underestimated the training-induced response in RTD but do not change the interpretation of the main findings in the current study.

In summary, the present study revealed that home-based, high-speed calf-raise training for 8 wk improves the rapid force-generating capability of the plantar flexion. The training-induced increment of the variables was accompanied by substantial changes in muscle activation of the trained muscles rather than musculotendinous factors. Although the training effect on standing balance performance was clinically trivial, the changes in triceps surae muscle activations during standing were substantial. Moreover, training-induced enhancement of the explosive plantar flexion strength at early phases was suggested to contribute beneficially to the standing balance performance. Such neuromuscular adaptations are expected to result in balance performance improvement. Our findings suggest that an improvement of functional capacity is induced after calf-raise exercises that can easily be performed at home by elderly people.

## GRANTS

Support for this study was partly provided by Japan Society for the Promotion of Science (JSPS) KAKENHI Grant Number JP25871206 [Grant-in-Aid for Young Scientists (B)].

## DISCLOSURES

No conflicts of interest, financial or otherwise, are declared by the authors.

## AUTHOR CONTRIBUTIONS

R.E., S.O., Y.K., and R.A. conceived and designed research; R.E., S.O., H.T., and R.A. performed experiments; R.E., S.O., H.T., and R.A. analyzed data; R.E., S.O., H.T., Y.K., and R.A. interpreted results of experiments; R.E. prepared figures; R.E. drafted manuscript; R.E., S.O., H.T., Y.K., and R.A. edited and revised manuscript; R.E., S.O., H.T., Y.K., and R.A. approved final version of manuscript.
